# TCTP Is a Critical Factor in Shrimp Immune Response to Virus Infection

**DOI:** 10.1371/journal.pone.0074460

**Published:** 2013-09-20

**Authors:** Wenlin Wu, Bingyan Wu, Ting Ye, Huagen Huang, Congjie Dai, Jianjun Yuan, Wei Wang

**Affiliations:** 1 Department of Biology, Quanzhou Normal University, Quanzhou, China; 2 College of Life Sciences, Zhejiang Sci-Tech University, Hangzhou, China; 3 The Third Institute of Oceanography, State Oceanic Administration, Xiamen, China; University of San Francisco, United States of America

## Abstract

The translationally controlled tumor protein (TCTP) is an abundant, ubiquitous, and conserved protein which plays important roles in a number of biological processes. In the present study, the TCTP in shrimp *Litopenaeus vannamei* was analyzed. The TCTP of *L.vannamei,* a 168-amino-acid polypeptide, shares a high degree of similarity with TCTPs from other species, having two TCTP protein signatures at the 45–55 aa and 123–145 aa motif. The mRNA and protein levels from different tissues were detected with the highest in muscle and the lowest in heart among all examined tissues. In addition, temporal TCTP expression was significantly up-regulated at 16 h and 48 h following infection with white spot syndrome virus (WSSV). Lastly, silencing of TCTP with dsRNA led to a significant increase of WSSV loads. These results provide new insights into the importance of TCTP as an evolutionarily conserved molecule for shrimp innate immunity against virus infection.

## Introduction

The translationally controlled tumor protein (TCTP) is initially described as a factor implicated in cell growth [1], [2]. It is a highly conserved 19-kDa protein, ubiquitously expressed in a wide range of eukaryotes, including yeast, plants, and animals [3]–[6]. Evidence indicates that TCTP plays important roles in a number of biological processes, such as cell growth [7], cell cycle progression [8], and anti-apoptotic activity [9]. Meanwhile, TCTP may be involved in response to extracellular signals and cellular conditions, such as calcium stress [10], starvation [11], heat shock [12], and heavy metals [13].

TCTP is also called Histamine Releasing Factor (HRF). It has been reported that all parasitic versions of TCTP/HRF proteins seem to be secreted into the vertebrate host organisms [14]. In addition, the malarial TCTP is a functional homologue of an immune mediator and can differentially modulate the secretion of cytokines, such as histamine from basophils and IL-8 from eosinophils, respectively [15]. In mice, filarial TCTPs may have a role in the allergic inflammatory responses to filarial infections [16]. In *Venerupis philippinarum*, the expression of TCTP was significantly decreased from 6 h to 12 h after infection, while it was up-regulated in that of 48 h [17]. All of these results suggest that TCTP may participate in a series of immune responses.

In recent years, *Litopenaeus vannamei*, as the main species of cultured shrimp, has been threatened by diseases, especially white spotsyndrome virus (WSSV), which have caused huge economic losses [18], [19]. Preventing and controlling the spread of WSSV has become a priority to the shrimp industry. Therefore, it is important to study the molecular mechanism underlying the shrimp *L. vannamei* immune responses against WSSV infection. In the present study, we reported investigation into the function of TCTP in the shrimp *L. vannamei* immune response against WSSV infection.

## Materials and Methods

### Shrimp Culture, WSSV Infection

Cultures of *L. vannamei* shrimp approximately 10 g and 10–12 cm each, were conducted in 80 L aquariums with air-pumped circulating seawater at 25°C [20], [21]. They were fed with commercial diet before and during experiments. WSSV inoculum was prepared from virus-infected *L. vannamei.* The tissues of infected shrimp were homogenized in TN buffer [20 mM Tris-HCl and 400 mM NaCl (pH 7.4)] at 0.1 g/mL. After centrifugation at 2000×g for 10 min, the supernatant was diluted to 1∶100 with 0.9% NaCl and filtered through a 0.45 µm filter. Then 0.1 mL of filtrate (10^5^ WSSV copies/mL) was injected into healthy shrimp in the lateral area of the fourth abdominal segment using a syringe. The 0.9% NaCl-injected shrimp was used as controls.

### Total RNA Isolation and cDNA Synthesis

Tissues from different organs were collected and total RNA was extracted with TRIzol reagent (Invitrogen), treated with DNaseI (Fermentas) and re-extracted with an equal volume of water-saturated phenol/chloroform (1∶1, v/v) followed by precipitation with 50% isopropanol. Total RNA (2 µg) was used for first strand cDNA synthesis using the PrimeScript Real-time PCR kit (TaKaRa) according to the user manual. Single-strand DNA was diluted to 1∶10 for subsequent SYBR Green assay.

### Transcription Analysis

The tissue distribution and temporal expression of TCTP (GenBank: EU305625.1) transcripts challenged with WSSV were determined by real-time PCR. The amplifications were conducted in triplicate in a total volume of 20 µL containing 10 µL of SYBR Premix EX Taq II (TakaRa, Japan). The TCTPF and TCTPR (as listed in Table 1) primers were used to amplify a fragment of 104 bp. VP28F and VP28R were used to amplify VP28 (GenBank: AF502435.1), a major viral envelope protein gene of WSSV. Two β-actin (GenBank: AF300705.2) primers, ActinF and ActinR were used to amplify a fragment of 105 bp as reference gene for internal standardization. The thermal profile for real-time PCR program was 95°C for 30 s, followed by 40 cycles of 95°C for 5 s, 65°C for 30 s. After the PCR program, data were analyzed with SDS 2.1 software (Applied Biosystems). For semi-quantitative RT-PCR, the PCR conditions were as follows: 94°C 2 min, followed by 30 cycles of 94°C 20 s,60°C 20 s, and 72°C 30 s for VP28, while 25 cycles for β-actin gene. The PCR products were analyzed on 2.0% agarose gel stained with ethidium bromide.

**Table 1:** Primers sequences for PCR amplification in this study.

Primers	Sequences
ActinF	5′-CCAGAGCAAGCGAGGTATCCTG-3′
ActinR	5′-CGTTGTAGAAAGTGTGATGCCAGA-3′
GFP-T7F	5′-taatacgactcactatagggATGGTGAGCAAGGGCGAGGA-3′
GFP-T7R	5′-taatacgactcactatagggTTACTTGTACAGCTCGTCCA-3′
TCTPF	5′-CAATGGACCCTGATGGC-3′
TCTPR	5′-GCTTCTCCTCTGTTAGACCGTAT-3′
TCTP-T7F	5′-taatacgactcactatagggATGAAGGTCTTCAAGGACAT-3′
TCTP-T7R	5′-taatacgactcactatagggTTATAGCTTCTCCTCTGTTAG-3′
VP28F	5′-TCCGCAATGGAAAGTCTGA-3′
VP28R	5′-TCAAAGGTGAGATTCTGCCC-3′

### Western Blot Analysis

The TCTP gene from *L. vannamei* was recombinantly expressed in *E. coli* BL21. The purified protein was used to immunize mice and the immunoglobulin (IgG) fractions were purified with protein A-Sepharose (Bio-Rad). The titers and specificity of the antibody were determined by an enzyme-linked immunosorbent assay (ELISA). Proteins of different tissues were extracted with Full Protein Extract Kit (Sangon, China) according to the protocol. Bradford-based method (Bio-Rad) was used to determine protein concentrations. Proteins were separated by SDS-PAGE, and then transferred to PVDF membrane. The membranes were blocked with 5% nonfat milk in Tris-buffered saline containing 0.1% tween-20 (TBS-T) overnight at 4°C, followed by incubation with appropriate primary antibody against TCTP (1∶500, or β-actin (1∶1000, Santa Cruz) for 2 h at room temperature. After washing 3 times for 10 mins each with TBS-T, the membranes were incubated with horseradish peroxidase-conjugated goat anti-mouse secondary antibody for 1 h. After extensive washing, labeling was visualized using the ECL Plus Western Blotting Detection System (GE Healthcare). The chemiluminescence signal was imaged using a ChemiDoc XRS (Bio-Rad) and quantified using Quantity One software (Bio-Rad).

### Synthesis of dsRNA

Double-stranded RNA (dsRNA) corresponding to the ferritin sequence was generated by *in vitro* transcription. DNA templates for dsRNA preparation were performed using the gene-specific primers with a T7 promoter sequence at the 5′ end. TCTP-T7F and TCTP-T7R. For an exogenous gene, a fragment of the green fluorescent protein (GFP) was amplified with the pEGFP-1 vector as template using GFP-specific primers. GFP-T7F and GFP-T7R. 1 µg of each template was used in *in vitro* transcription using the T7 RiboMAX Express Large Scale RNA production Systems (Promega, USA) according to the manufacturer’s instructions. The remaining DNA template in the solution was digested with RNase-free DNase I. Then, the dsRNA was purified with TRIzol reagent (Invitrogen) following the protocol. Before injection into shrimp, dsRNA was verified by treatment with RNaseA and RNaseIII. Finally, the concentration of each dsRNA was estimated using spectrophotometry at an absorbance of 260 nm.

### *In vivo* Gene Silence

To silence the expression of shrimp TCTP, the dsRNAs were injected into shrimp according to Amoaryup [22] and Huang [23]. Briefly, 10 µg (low concentration) or 20 µg (high concentration) of TCTP dsRNA dissolved in 100 µl of 150 mM NaCl was intramuscularly injected, with 20 µg GFP dsRNA as negative control. Shrimp was injected every day for 4 days. At the second injection, WSSV (10^4^ copies/shrimp) and dsRNA were injected together.

### WSSV Quantification

To quantify WSSV in shrimp, qRT-PCR was conducted using WSSV-specific primers and a TaqMan fluorogenic probe with a WSSV qRT-PCR kit (LLBio, China) according to the manufacturer’s instructions and Liu [24]. The linearized plasmid containing a DNA fragment from the WSSV genome was used as an internal standard for qRT-PCR. For each treatment, 20 individual shrimp were used and gill tissues were collected at 48 h post-inoculation. Three shrimp specimens were selected at random from each treatment and were subjected to qRT-PCR to quantify WSSV genome copies. Virus genomic DNA was extracted from shrimp gills. The PCR mixture (20 µl) contained 18 µl reaction buffer and 2 µl DNA template. The reaction profile was 95°C for 2 min, followed by 40 cycles of 10 s at 94°C, 30 s at 60°C. After the PCR program, data were analyzed with SDS 2.1 software (Applied Biosystems).

### Sequence Analysis and Phylogenetic Analysis

The TCTP gene sequence was analyzed using the BLAST algorithm at NCBI web site (http://www.ncbi.nlm.nih.gov/blast), and the deduced amino acid sequence was analyzed with the Expert Protein Analysis System (http://www.expasy.org). Amino acid sequences from other organisms were retrieved from NCBI GenBank. DNAMAN software version 6.0 was used to analyze multiple alignments. Phylogenetic analysis was performed with Clustal and the Neighbor-joining (NJ) algorithm of MEGA 4.0.

### Statistical Analysis

One-way analysis of variation (ANOVA) was used to analyze the data from three independent experiments. Statistical significance between treatments was carried out using the Student’s *t*-test. Differences were considered statistically significant at P<0.05.

## Results

### Characterization of *L. vannamei* TCTP

**Figure 1:**
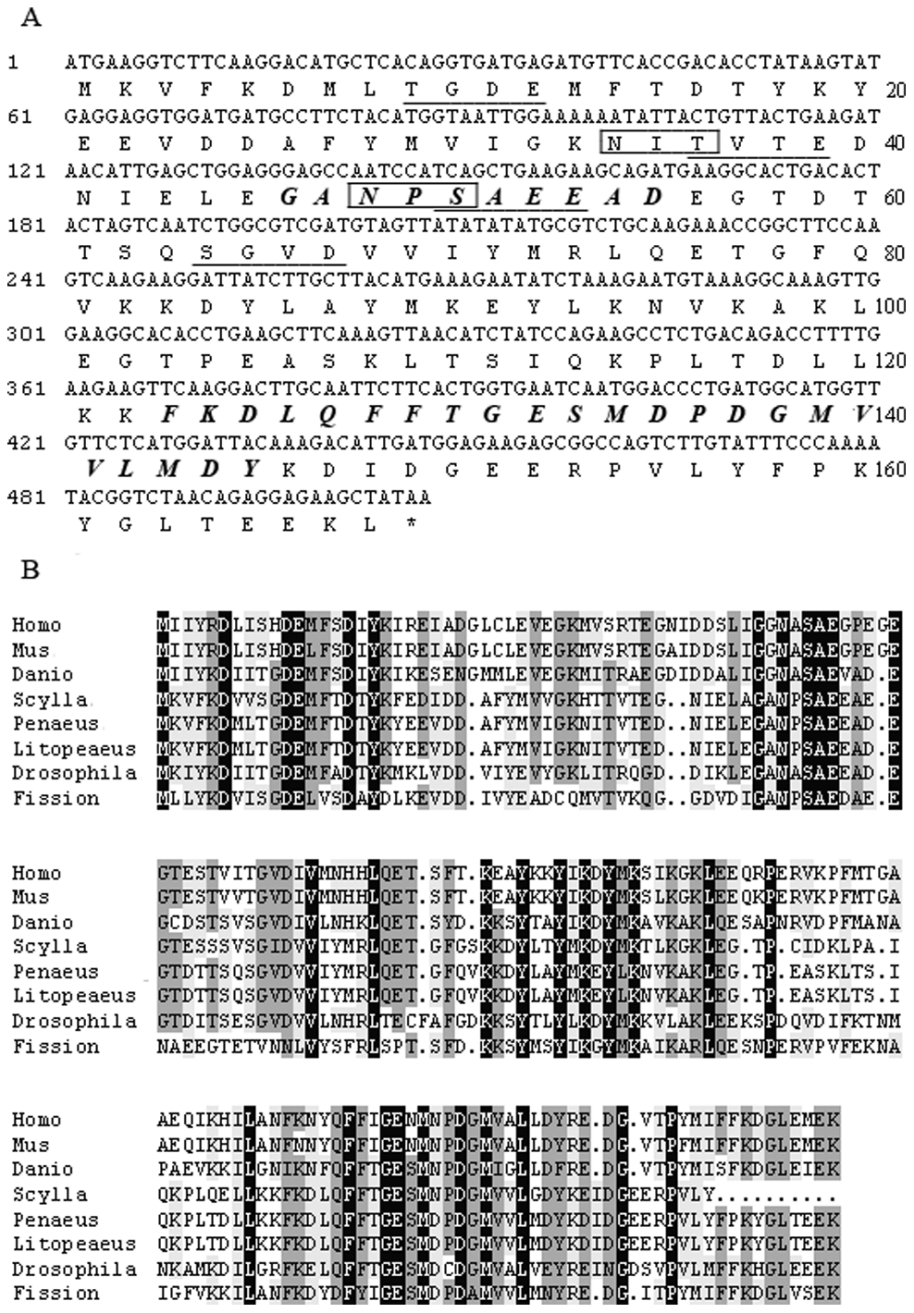
Characterization of *Litopeaeus vannamei* TCTP. A) The *Litopeaeus vannamei* TCTP ORF, the deduced amino acid sequence, and the motif analysis. The highly conserved TCTP signature 1 and 2 sequences are shown in bold italic letters. Four CK2-phospho-sites (casein kinase II phosphorylation site) are underlined. Two potential Asn-glycosylation sites are shown in textbox. B) TCTP alignment. The following species were aligned: *Homo sapiens* (NP_003286.1), *Mus musculus* (P14701), *Danio rerio* (NP_937783.1), *Scylla paramamosain* (ACY66461), *Penaeus monodon* (ACD13588.1), *Litopeaeus vannamei* (ABY55541.1), *Drosophila melanogaster* (AAF54603.1), *Fission yeast* (Q10344).

The *L. vannamei* TCTP, a-168-amino- acid polypeptide, is highly homologous to known TCTP proteins at the 45–55 aa and 123–145 aa motif, which are TCTP protein signatures (Fig. 1A). *L. vannamei* TCTP contained a potential Asn-glycosylation site (N-glycosylation site) at 34–37 aa (NITV); four CK2-phospho-sites (casein kinase II phosphorylation site) at 9–12 aa (TGDE), 36–39 aa (TVTE), 50–53 aa (SAEE), and 64–67 aa (SGVD); one N-myristoylation sites at 46–51 aa (GANPSA) (Fig. 1A).

To elucidate the conservation of TCTP, multiple alignments of amino acid sequences were conducted with other species, each representing one kingdom. The result showed that twenty-nine of the approximately 170 residues comprised a total of nearly 17% absolutely conserved amino acids. The amino acid sequence encoded by L. vannamei shared 100%, 72.6%, 57.6%, 44.8%, 41%, 42.8%, and 39.8% identities with *Penaeus monodon*, *Scylla paramamosain*, *Drosophila melanogaster*, *Diano rerio*, *Homo sapiens*, *Mus musculus*, and *Fission yeast*, respectively (Fig. 1B).

**Figure 2:**
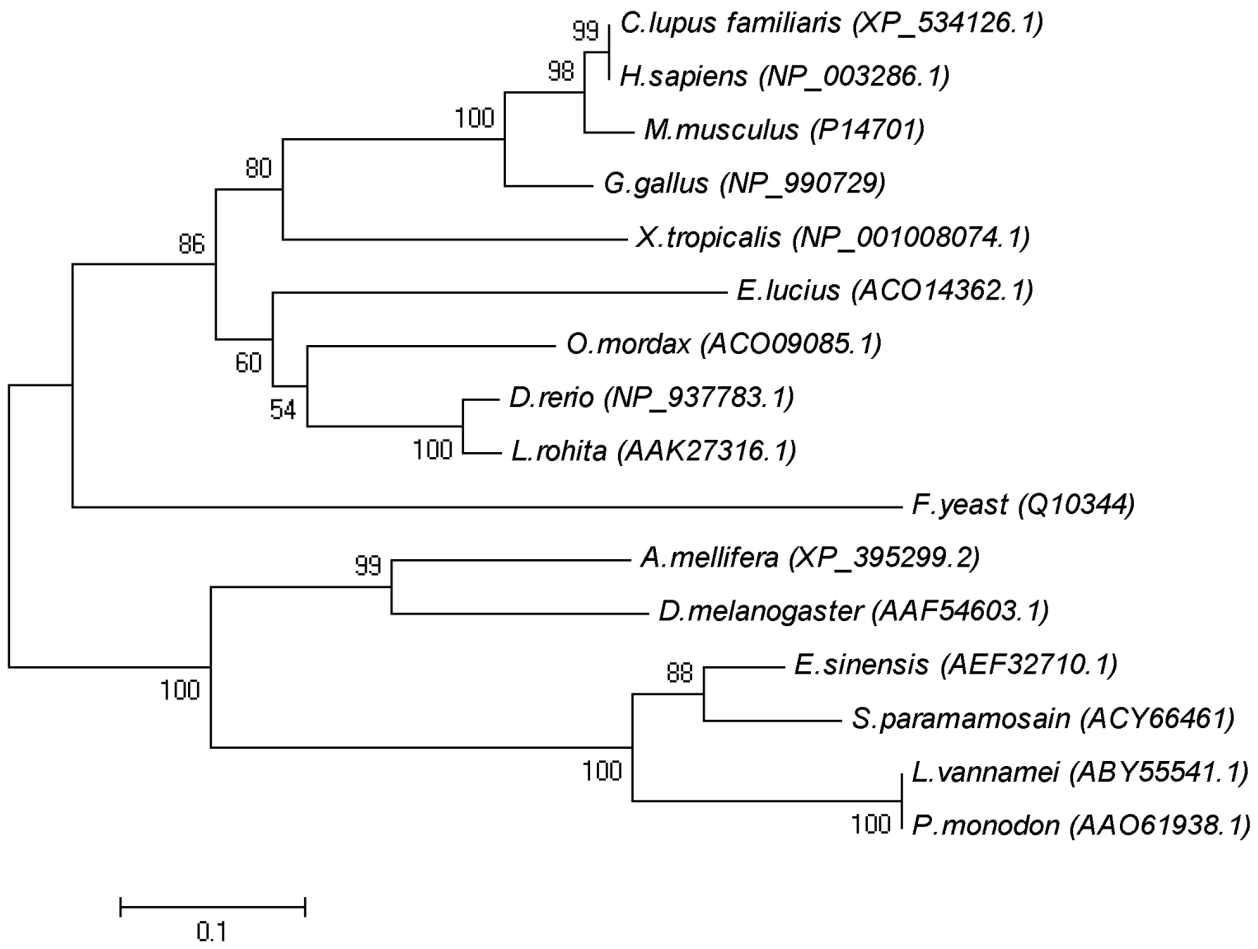
Phylogenetic tree of the *L. vannamei* TCTP protein. The tree was constructed by the MEGA 4.0 program. Bootstrap analysis was performed using 1000 replicates to test the relative support for particular clades. Scale bar means evolutionary distance, and it was 0.1 million years.

Phylogenetic relationship analysis revealed that the deduced amino acid sequence of *L. vannamei* TCTP was in the same distinct branch of invertebrates. This branch was also included other arthropods, such as the fruit fly and honey bee. Phylogenetic analysis also revealed that *L. vannamei* TCTP constitutes a well-defined subgroup within the TCTPs of other crustaceans with high bootstrap values (Fig. 2).

### Expression Patterns of *L. vannamei* TCTP

**Figure 3:**
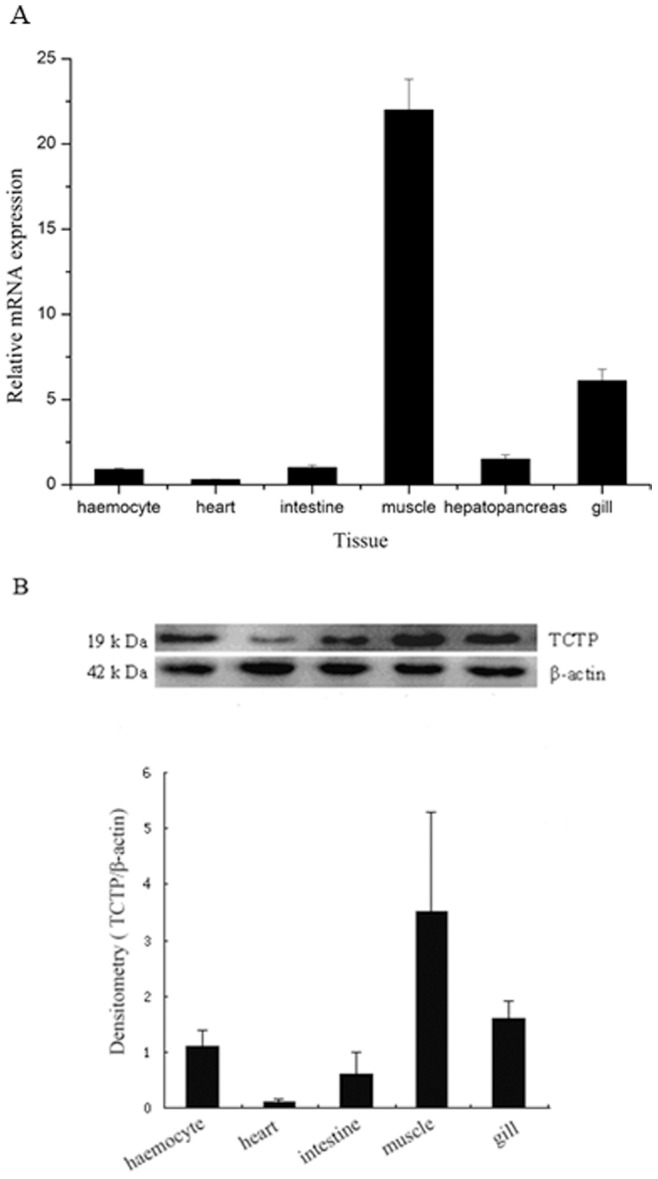
Expression profiles of *L. Vannamei* TCTP. A) Relative expression of TCTP in different tissues of *Litopeaeus vannamei* determined by Real-time RT-PCR. Total RNA was extracted from haemocyte, heart, intestine, muscle, hepatopancreas, and gill of healthy shrimp respectively. B) Protein level of TCTP in different organs. Protein level was analyzed by Western blot. Loading of the lanes was normalized to levels of β-actin and the experiment is representative of five independent experiments.The calculated densitometry intensities of the respective bands were present as fold of β-actin. The results are expressed as means ± S.D (n = 5).

TCTP transcripts from different tissues were determined by real-time PCR to examine the tissue distribution. As shown in Fig. 3A, TCTP mRNAs were detected in all target tissues at different levels. The expression of TCTP transcript was the highest in the muscle and the second-highest in gills. The levels of TCTP in intestine and haemocyte were similar to each other, but lower than that of hepatopancreas. The expression level in heart was the lowest. To further validate the results of real-time PCR, the protein levels of TCTP were detected by Western blot with *L. vannamei* TCTP polyclonal antibody (Fig. 3B). Because of the post-transcriptional regulation and post-translational modification, the protein levels were not the same as mRNA, but the same pattern of protein levels was observed in all examined tissues except hepatopancreas, with the highest in muscle and the second-highest in gills. Due to the high activity of proteinase, the protein in hepatopancreas was degraded quickly and was unable to be detected by Western blot.

### Temporal Expression Profiles of TCTP Post WSSV Challenge

**Figure 4:**
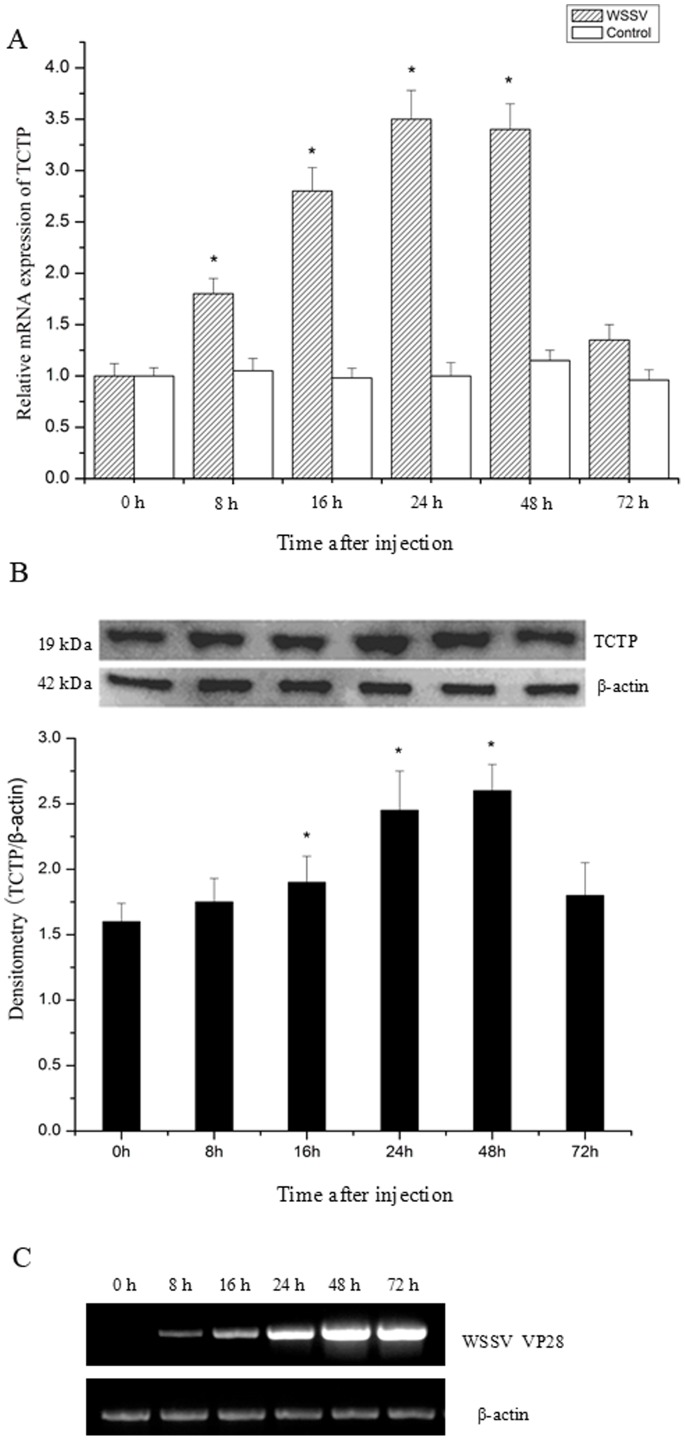
Temporal expression level of TCTP in gills after WSSV infection. A) Transcripts determined by real-time RT-PCR at different time post WSSV infection. B) TCTP protein level determined by Western blot. Loading of the lanes was normalized to levels of β-actin and the experiment is representative of three independent expriments. The calculated densitometry intensities of the respective bands were present as fold of β-actin. The results are expressed as means ± S.D (n = 3). The asterisk indicates that the expression levels are significantly different (P<0.05). C) Semi-quantitative RT-PCR expression profiles for transcripts encoding β-actin and VP28 of WSSV during time-course infection of WSSV.

To characterize TCTP gene during virus infection, the temporal expression of TCTP in gills after WSSV challenge was determined by real-time PCR with β-actin as internal control. TCTP transcript was up-regulated at 8 h (1.8-fold), 16 h (2.8-fold), 24 h (3.5-fold), and 48 h (3.4-fold) post WSSV injection and dropped to the basal level at 72 h (Fig. 4A). However, no significant increase in TCTP gene expression was observed in the shrimp injected with 0.9% NaCl as control. These results were further confirmed by Western blot (Fig. 4B). In the same time, WSSV was detected in gills at 8 h post injection and increased quickly as shown by the semi-quantitative PCR results (Fig. 4C).

### Effects of TCTP on Shrimp Antiviral Immunity

**Figure 5:**
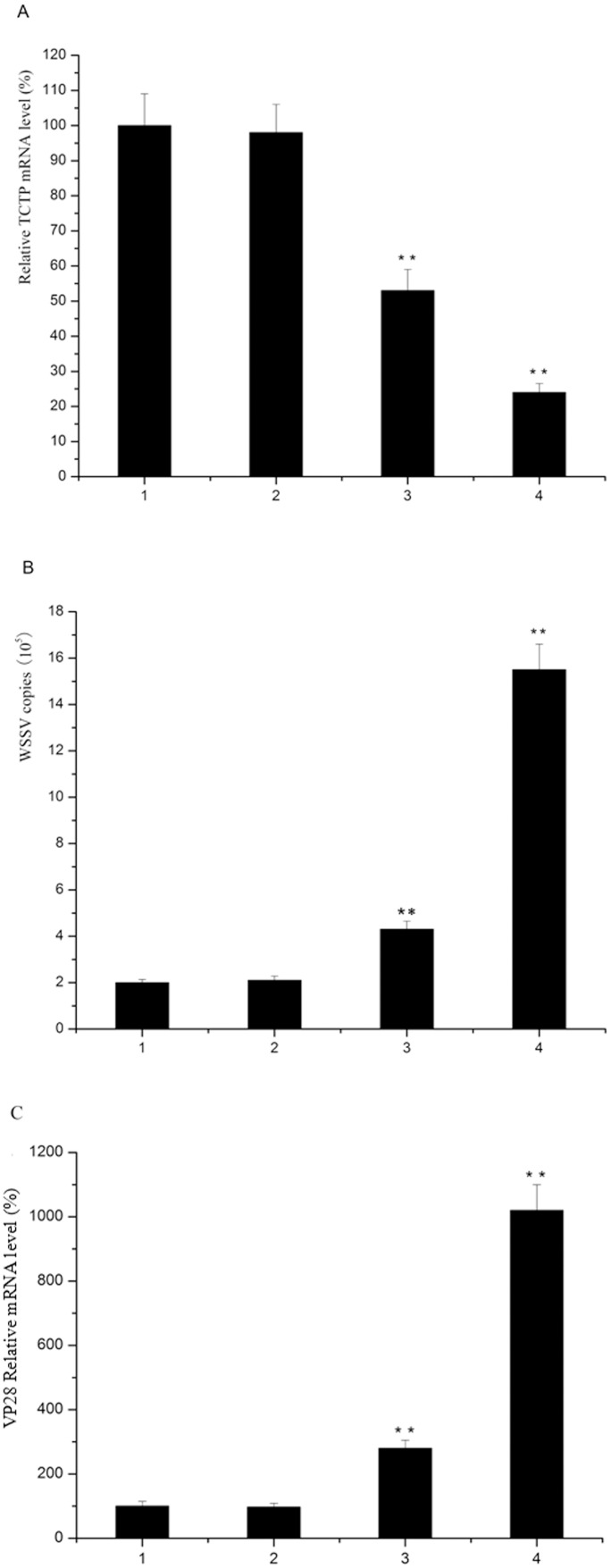
Roles of TCTP in the shrimp immune response against WSSV infection. A) Shrimp were injected with TCTP, GFP dsRNA or NaCl. Gills from each shrimp were collected 48 h post challenge for real-time PCR to detect the specific of dsRNA. B) Shrimp were injected with TCTP, GFP dsRNA or NaCl every day for 4 days. At the second injection, WSSV (10^4^ copies/shrimp) and dsRNA were injected together. At 48 h post infection, the shrimp from each treatment were subjected to quantitative real-time PCR to quantify WSSV copies. C) WSSV VP28 transcripts were determined by real-time PCR. 1, 2, 3, 4 represent shrimps injected with NaCl, GFP dsRNA, 10 µg TCTP dsRNA and 20 µg TCTP dsRNA, respectively. The asterisk indicates the statistical significantly different (**P<0.01).

To investigate the role of TCTP in antiviral immunity, we first used dsRNA to silence the expression of TCTP in shrimp. Shrimp were injected with 10 µg or 20 µg TCTP dsRNA and the mRNA levels of TCTP transcript were determined 2 days after injection. The results of real-time PCR showed that the mRNA level of TCTP was knocked down by approximately 75% in high concentration (20 µg/shrimp) and by approximately 45% in low concentration (10 µg/shrimp), whereas injection of NaCl or GFP dsRNA had no significant effect on the TCTP transcript levels. (Fig. 5A). These data revealed that dsRNA could efficiently silence the expression of TCTP.

Then, WSSV infection in shrimp was evaluated after TCTP silencing. Shrimp were challenged with WSSV after the expression of TCTP was silenced using TCTP specific dsRNA. In TCTP silence group, white spots first appeared on the carapace and the fifth-sixth abdominal segments of the shrimp 24 h post WSSV injection, and later on the shell of the whole body. The shrimp reduced feed intake or stopped feeding, followed by reduction in preening activity and locomotion, which were manifested by gill fouling and a weak response to stimulation. More moribund shrimps were found in TCTP silence group compared with the control 48 h post WSSV injection, and they stayed at the pond edge or swam slowly at the water surface before finally sinking to the pond bottom. The amount of virus in the shrimp was analyzed and compared with the control. Low levels of viral infection were observed in control shrimp (no dsRNA) and in shrimp with non-specific dsRNA (GFP). WSSV copies in shrimp treated with TCTP dsRNA were significantly different from the control. Knockdown of TCTP led to approximately 3-fold (low concentration dsRNA) and 8-fold (high concentration dsRNA) increase in viral load in the treatment (WSSV+TCTP dsRNA) compared with the control (P<0.01) (Fig. 5B). To further confirm these results, the levels of VP28 were detected by real-time PCR. The results indicated that the expression level of VP28 in TCTP silenced shrimp was much higher than that of the control (Fig. 5C). These data suggested that the silence of TCTP led to the increase of viral replication, indicating that TCTP played important roles in the host immune response against virus infection.

## Discussion

Diseases caused by viruses, especially by white spot syndrome virus (WSSV), are the greatest challenge to shrimp aquaculture. A better understanding of shrimp immune response will be helpful for disease control. Although TCTP play an important role in the anti-stress program of many organisms, its roles in immune response is still remained limited, particularly in invertebrates. In this study, we reported the molecular characterization of TCTP from shrimp *L. vannamei* and its roles in antiviral immune responses.

As revealed in the study, the *L. vannamei* TCTP shared high similarities with other species, suggesting that the function of TCTP is conservative. The TCTP gene was constitutively expressed in various tissues examined. Real-time PCR showed that the expression of TCTP was highest in muscle and lowest in heart. This may be due to TCTP roles involved in growth-related activities [25] and muscle is a quick-growing tissue, but heart grows slowly. The expression level of TCTP in gills was the second-highest. Gills are in direct contact with the external environment, therefore, their cells are crucial in response to external biotic and abiotic factors. In shrimp, gills play an important role in defense. For example, gills were the site of formation of hemocyte nodules during foreign particle injection [26] and accumulation of viable bacteria during infection [27]. Due to the high expression of TCTP and their important roles in immune activities, gills were selected as the detection target in this study.

The present study indicates that the expression of TCTP was up-regulated after WSSV challenge in both mRNA and protein level, suggesting that TCTP might play an important role in the immune response of the shrimp. To further study the function of TCTP in shrimp innate immunity, dsRNA was used to silence the expression of TCTP before WSSV challenged. It was revealed that the knockdown of TCTP by dsRNA significantly increased viral loads, indicating that TCTP was involved in shrimp immune responses against WSSV infection.

It has been reported that viruses can activate various host signaling pathways mediated by some small GTPases to modulate microtubule-based transport of virus particles from the plasma membrane to the nucleus during the establishment of infection [28], [29]. TCTP is thought to be a modulator of GTPase activity, acting as a molecular switch for a vast number of cellular processes in all eukaryotes [30]. TCTP has been reported to bind to the nucleotide-free form of Rab proteins [31]. In our previous report, Rab was identified to compose a complex with actin, tropomyosin, and WSSV protein, VP466, in which the Rab GTPase might detect virus infection as an intracellular virus recognition protein and trigger downstream phagocytic defense against virus in shrimp [21]. The present study indicated that TCTP was up-regulated after WSSV challenge. Taken together, we speculated that TCTP may be included in the phagocytosis against virus by interacting with Rab GTase.

TCTP has been demonstrated to be a potent anti–apoptotic protein by binding interactions with MCL1 [32] and BCL-XL [33], Caspase-3 [34]. In some cell lines, up-regulating cellular TCTP levels led to cellular protection against cell death induced by oxidative stress [35], [36]. During the combat between host and pathogen, respiratory burst is a key stagy to eliminate the pathogen. This may lead to oxidative stress induced by reactive oxygen species (ROS) and cause cell death. This may require the up-regulation of the expression of anti-apoptotic protein TCTP to minimize the negative effects.

The expression of TCTP was up-regulated post WSSV injection. Silencing TCTP significantly increased virus load compared to control shrimp, indicating that TCTP might play important roles in the host defense against virus infection. This study provided new insights into the role of TCTP in the immune response of invertebrates against virus infection.
