# Correction: TCTP Is a Critical Factor in Shrimp Immune Response to Virus Infection

**DOI:** 10.1371/journal.pone.0116331

**Published:** 2014-12-17

**Authors:** The PLOS ONE Staff

The *PLOS ONE* editorial office was made aware that prior work by Prof. Amornrat Phongdara and colleagues on the role of fortilin/TCTP in response of the shrimp *Penaeus monodon* to viral infections were not cited in this article. After consulting with Academic Editors, the *PLOS ONE* staff editors believe that these articles should have been cited. These articles are:

Tonganunt M, Nupan B, Saengsakda M, Suklour S, Wanna W, Senapin S, Chotigeat W, Phongdara A: The role of Pm-fortilin in protecting shrimp from white spot syndrome virus (WSSV) infection. Fish Shellfish Immunol. 2008 Nov;25(5):633-7. doi: 10.1016/j.fsi.2008.08.006.

Nupan B, Phongdara A, Saengsakda M, Leu JH, Lo CF: Shrimp Pm-fortilin inhibits the expression of early and late genes of white spot syndrome virus (WSSV) in an insect cell model. Dev Comp Immunol. 2011 Apr;35(4):469-75. doi: 10.1016/j.dci.2010.11.016.

Panrat T, Sinthujaroen P, Nupan B, Wanna W, Tammi MT, Phongdara A: Characterization of a novel binding protein for Fortilin/TCTP—component of a defense mechanism against viral infection in Penaeus monodon. PLOS ONE 2012;7(3):e33291. doi: 10.1371/journal.pone.0033291.

The authors apologize for not citing this work, and have provided an updated discussion at http://www.plosone.org/annotation/listThread.action?root=74639
